# Allopatric molting of Devonian trilobites

**DOI:** 10.1038/s41598-022-18146-3

**Published:** 2022-08-16

**Authors:** Ruiwen Zong, Yiming Gong

**Affiliations:** State Key Laboratory of Biogeology and Environmental Geology, China University of Geosciences, Wuhan, 430074 China

**Keywords:** Animal behaviour, Palaeontology, Palaeontology

## Abstract

Trilobite exuviae record the development of individual trilobites and their molting process and can also contain information on their behavior. The silt- to fine-grained tuffites of the middle part of the Middle Member of the Upper Devonian Hongguleleng Formation in western Junggar contains abundant phacopidae trilobite, specifically *Omegops* sp. A, almost all of which are exuviae. Based on the preservation pattern, burial environment, and set of organisms co-occurring with *Omegops* sp. A, we speculate that the environment represented by the middle part of the Middle Member of the Hongguleleng Formation served only as the molting site of *Omegops* sp. A, and that their primary habitat was elsewhere. *Omegops* sp. A would have thus travelled to deep-water to molt. The reasons for allopatric molting may have included avoiding predators and interference from competing organisms during molting. This implies that the migratory behavior of some modern arthropods may have existed since at least the Devonian. This behavior suggests that Late Devonian phacopidae trilobites may have migrated to deeper water expanded their ecological domain as a survival strategy in response to unfavorable ecological environment.

## Introduction

Like modern arthropods, trilobites had to molt periodically as they grew^1^. Trilobites underwent several molts during their lifetimes, and the exuviae can be found as fossils. This is one reason for the high number of trilobite fossils found in the Paleozoic strata. In addition to reflecting the growth process of trilobites and morphological changes during individual development, some specimens can, however, reveal certain morphological or preservational aspects from which behaviour or survival strategies may be inferred. For example, exuviae found in the shells or burrows of other organisms suggest that molting trilobites would seek shelter^2–5^. Large collections of exuviae are interpreted as evidence that some trilobites molted in aggregations, indicating collective behavior^6,7^. There is also evidence that some would burrow into the sediment to molt, reflecting an adaptive evolution to predation and high-stress ecological environments^8^.

Different trilobites have different molting processes, which have been identified and described for numerous trilobite species^1,9–11^. For the phacopidae trilobites, their librigenae were fused with the cranidium, which resulted in a different molting process than most trilobites, that is, separation of the cephalon from the thoracopygon usually occurred during molting. The scattered cephalon and articulated thorax and pygidium preserved within the strata are thus widely considered as phacopidae exuviae^10^. The Late Devonian Famennian strata of western Junggar, Xinjiang, contain a large number of phacopidae trilobite exuviae. In this paper, we explore the possible behavioral significance of the exuviae based on the preservation pattern and burial environment of these specimens.

## Geological setting, materials and methods

**Figure 1:**
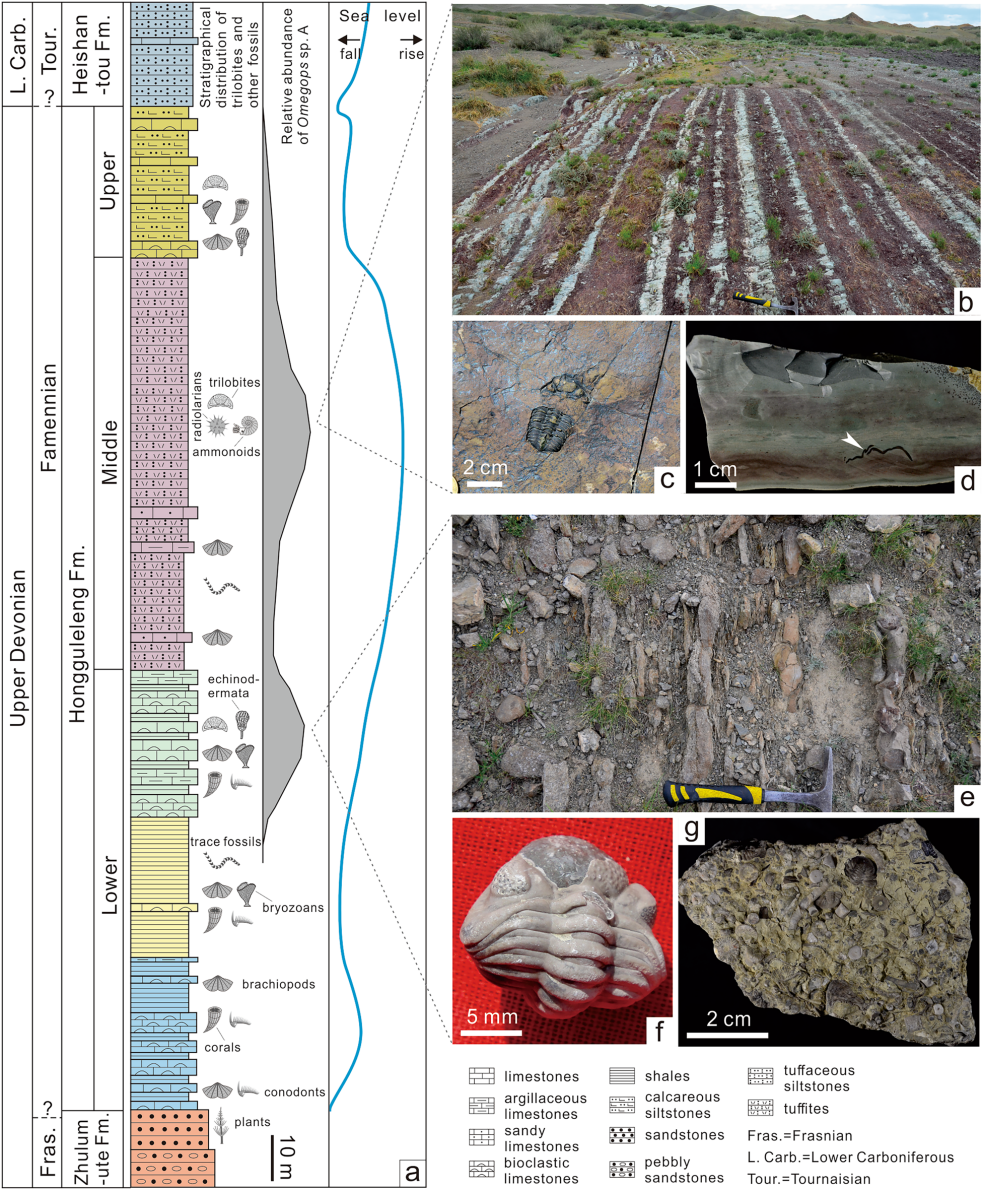
Comprehensive stratigraphical column of the Hongguleleng Formation, and trilobite-bearing tuffites and bioclastic limestones in western Junggar. (**a**) Stratigraphical distribution of *Omegops* sp. A and other fossils in the Hongguleleng Formation and the Late Devonian sea-level change curve in the study area. (**b**) Silt- to fine-grained tuffites in the middle part of the MHF in the Bulongguoer section. (**c**) Exuviae of an *Omegops* sp. A preserved on the bedding surface of the tuffites. (**d**) Vertical section of the trilobite-bearing tuffites. (**e**) Thin-bedded bioclastic limestones, argillaceous limestones and shales unevenly interbedded in the upper part of the LHF. (**f**) enrolled *Omegops* sp. A from the upper part of the LHF. (**g**) Bioclastic limestones contain trilobite fragments and other bioclasts.

Western Junggar is an important part of the Central Asian Orogenic Belt, and composed of a series of Paleozoic volcanic arcs and accretionary complexes^12,13^. Devonian and Carboniferous strata are widely distributed in this area, among them, the Famennian (Late Devonian) strata, represented by the Hongguleleng Formation, can be divided into three members from the bottom to top^14^. The Lower Member (LHF) is composed of bioclastic limestones, shelly limestones, argillaceous limestones, calcareous siltstones, and shales. It can be further divided into three parts, the lower part is dominanted by shelly limestones; the middle part is composed of calcareous siltstones and a small amount of thin-bedded argillaceous limestones in its stratotype section (Bulongguoer section), however, this part often changes to argillaceous limestones or shell-bearing sandy conglomerates in other stratal sections; the upper part is the uneven interbedded bioclastic limestones, argillaceous limestones and shales. The LHF was deposited in an environment with the influence of storms. The Middle Member (MHF) comprises grayish-green and grayish-purple volcaniclastic rocks and a few sandy limestones, it can also be roughly divided into three parts, the lower part is mainly composed of the fine- to medium-grained tuffites, with a few thin-bedded sandy limestones or argillaceous limestones; the middle part is dominanted by silt- to fine-grained tuffites, occasionally interspersed with less than 1 cm thick medium-grained tuffites; the fine- to medium-grained tuffites slightly increased to the upper part. The Upper Member (UHF) is primarily grayish-yellow calcareous clastic rocks with a few bioclastic limestones^15–17^. The Hongguleleng Formation was emplaced in a retroarc basin^16^, in which over time the water column gradually deepened and then became shallow from the lower part to middle part of the LHF, and gradually deepened again since the upper part of the LHF, then become dramatically shallower in the UHF^18^ (Fig. 1a). The Hongguleleng Formation contains abundant fossils, especially in the Lower Member, such as brachiopods, corals, echinoderms, trilobites, bivalves, gastropods, ostracods, conodonts, chondrichthyans, bryozoans, cephalopods, radiolarians, and trace fossils^19^.

Trilobites are found in all three members of the Hongguleleng Formation^17,20–22^, most abundantly in the bioclastic limestones and argillaceous limestones of the upper part of the LHF and in the silt- to fine-grained tuffites of the MHF (Fig. 1). However, the diversity of trilobite species is very low; only three have been identified: *Omegops accipitrinus mobilis* (Xiang), *Omegops cornelius* (Richter and Richter), and *Phacops circumspectans tuberculosus* Yuan and Xiang^20,21^. Recently, we restudied these three species based on a large number of specimens collected from the same horizon, the former two species are probably two new species, which are tentatively named *Omegops* sp. A. and *Omegops* sp. B. The last species was established only based on an incomplete cephalon^21^. We re-examined this specimen and found that it might also be *Omegops* sp. A. The *Omegops* sp. A was distributed from the upper part of the LHF to the top of the Hongguleleng Formation, while *Omegops* sp. B was only distributed in the upper part of the LHF (our unpublished materials). The trilobites in the Hongguleleng Formation have been found in three preservation patterns. The first is enrolled or articulated exoskeletons, which are entire fossilized carcasses, but this type is rare and mainly found in the LHF. Most of the fossils are scattered sclerites, specifically an attached thorax and pygidium, with a detached cephalon nearby, and hypostome is occasional near the thorax. These are likely empty shells shed by molting trilobites. The third type found is disarticulated cephala, thoraces, and pygidia, or scattered thoracic segments, which are probably trilobite carcasses or exuviae that were transported by a bottom current before burial, or carcasses destroyed by scavengers. These three types co-occurred in the LHF. However, in the MHF, especially in the middle part of this member, the first type is very rare, almost all trilobites are belong to second type; the third type is found rarely in the fine- to medium-grained tuffites of the lower part of the MHF.

The materials in this study were collected from the silt- to fine-grained tuffites of the middle part of the MHF in Wulankeshun and Bulonguoer sections of western Junggar^17^. Trilobites are very abundant in the tuffites: since 2010, we have collected more than 1000 trilobites from the middle part of the MHF from both two sections, of which 123 were specimens collected and stored in the State Key Laboratory of Biogeology and Environmental Geology, China University of Geosciences, Wuhan (BGEG); the other 889 samples were data or photos collected in the field. These trilobites were all *Omegops* sp. A, no other trilobites were found in the same horizon.

**Figure 2:**
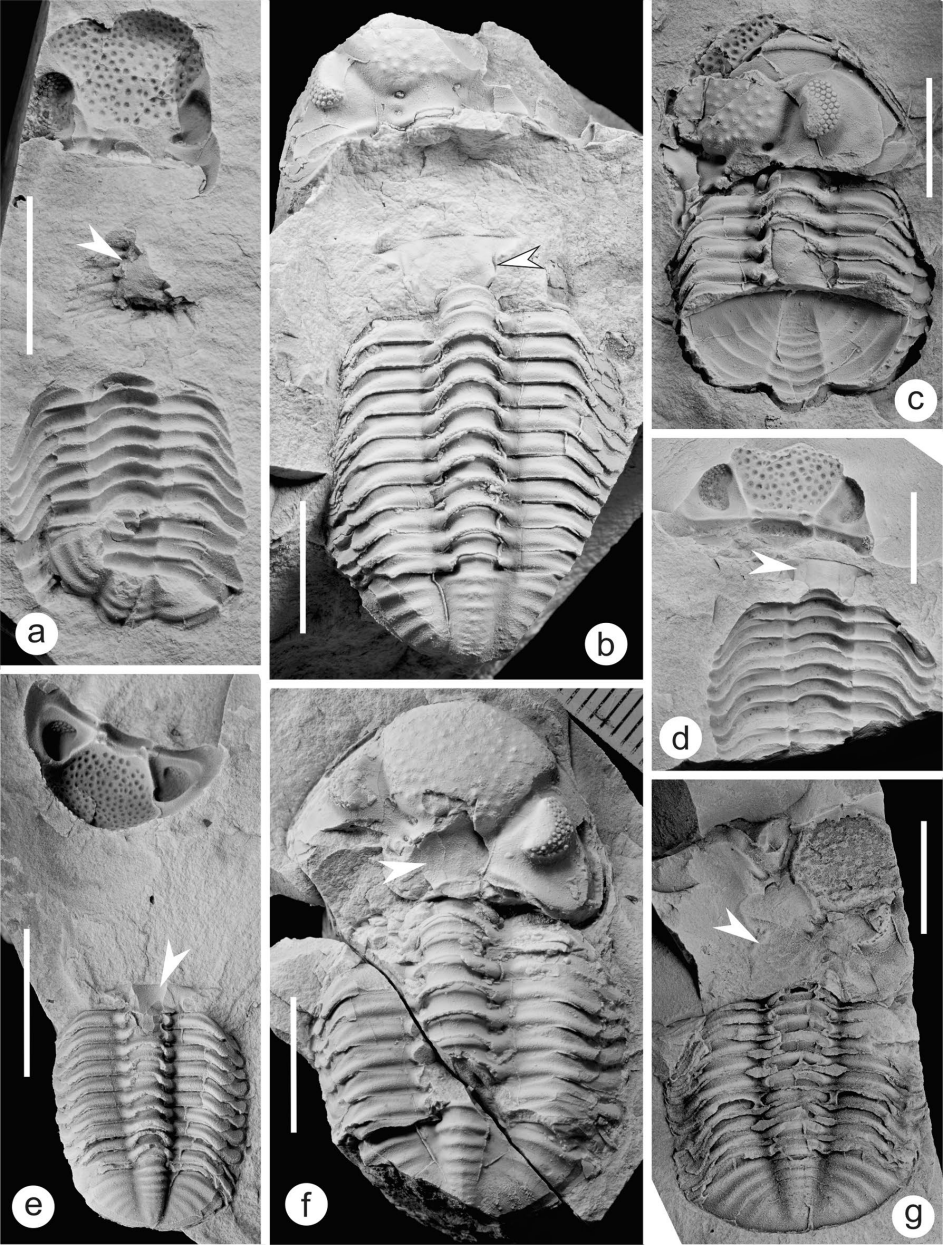
Different types of exuviae of *Omegops* sp. A from the middle part of the MHF in western Junggar. (**a**) Enrolled thoracopygon with separated cephalon, external mold (BGEG-WJ18-02). (**b**) Flat extended thoracopygon with separated cephalon (BGEG-WJ18-03). (**c**) Separated cephalon overlapping enrolled thoracopygon (BGEG-WJ18-05). (**d**) Cephalon separated from thorax, pygidium is not preserved, external mold (BGEG-WJ18-01). (**e**) Flat extended thoracopygon, with separated inverted cephalon (BGEG-WJ18-06). (**f**) Cephalon separated from thoracopygon and rotated at a small angle (BGEG-WJ18-04). (**g**) Cephalon separated from thoracopygon and rotated at a large angle (BGEG-WJ-77). Scale bar is 10 mm, white arrows indicate the position of the hypostomes.

Because the tuffites of the middle part of the MHF is very hard, therefore, in the field sampling, we first look for trilobite fossils at the surface of tuffites. For easily stripped tuffites, geological hammers are used to split the layers to look for trilobites preserved inside the rocks. Those trilobites that could be recovered were brought back to the laboratory, and those that could not be recovered, their preservation patterns were recorded in the field, including whether the cephalon, thorax and pygidium are articulated, whether the thorax is inverted, whether the hypostome is visible, and whether there are co-occurring macrofossils. The fossils in Fig. 2 were whitened with magnesium oxide powder, and all photographs were captured using a Nikon D5100 camera with a Micro-Nikkor 55 mm F3.5 lens.

## Results

### Preservation pattern of the trilobites

**Figure 3:**
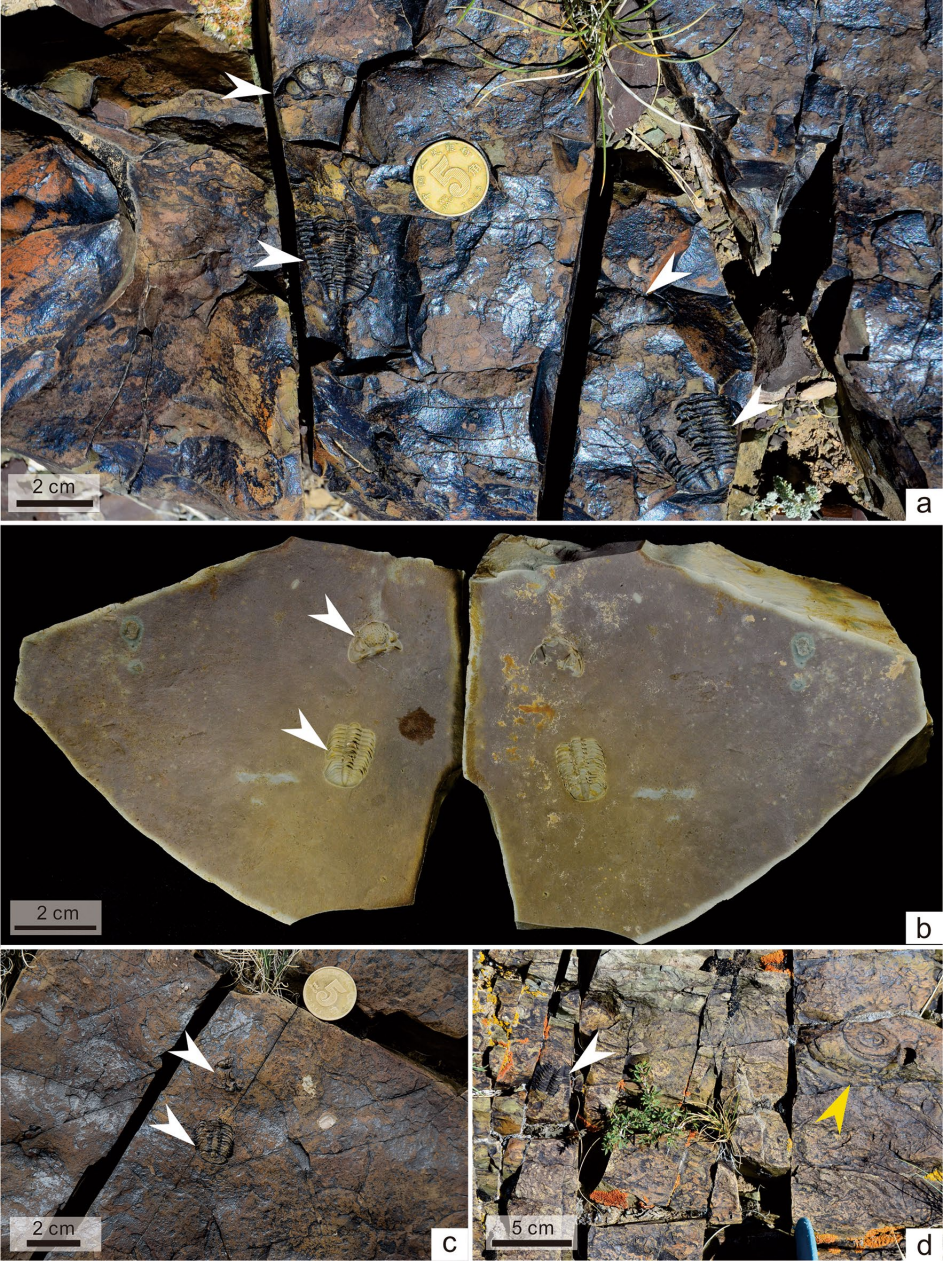
*Omegops* sp. A (white arrows) preserved in the tuffites of the middle part of the MHF, with no other benthic fossils in the same beds. (**a, c**) from the Wulankeshun section and (**b**) from the Bulonguoer section (BGEG-WJ18-07). (**d**) *Omegops* sp. A and ammonoids (yellow arrow) on the bedding surface of tuffites. (**a**, **c**, and **d**) are field photos.

Of the more than 1000 specimens of *Omegops* sp. A we have collected, fewer than 10 are articulated exoskeletons (specifically, seven unenrolled and two enrolled exoskeletons). All the rest are separated cephala with articulated thoracopyga (Figs. 2, 3), but no disarticulated thorax and pygidium. There is no contact or queuing between separate individuals, though in places two individuals were preserved several centimeters apart (Fig. 3a). Although there is some variation in their size (about 1.5 to 4 cm long), all specimens are holaspid individuals; no example of individuals in the protaspid or meraspid stages have yet been found. The more complete specimens clearly show that the separated rotated or inverted cephala are preserved in close proximity to the articulated flat or enrolled thoracopyga, with the convex side of the thorax facing upward. In addition, on some specimens, hypostomes can be seen on the anterior side of the thorax (Fig. 2). However, there are still a large number of specimens where the hypostomes may have been buried in the surrounding rock, covered by the thoraxes or cephala, or even erased by weathering, so that they cannot be seen. In general, this preservation pattern, i.e., the rotated or inverted cephalon is separated from the thoracopygon, the pygidium articulated with thorax that the convex side facing upward, is consistent with patterns attributed to phacopidae exuviae in previous studies^1,10,11^.

### Burial environment of *Omegops* sp. A from the middle part of the MHF

**Figure 4:**
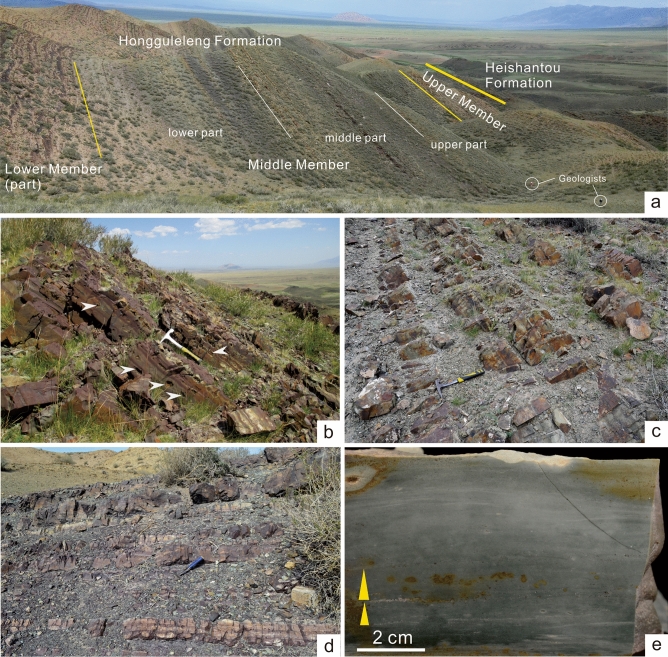
Characteristics of the lithological assemblage in the Middle Member of the Hongguleleng Formation in Western Junggar. (**a**) Outcrop of the Middle Member of the Hongguleleng Formation in the Wulankeshun section. (**b**) Tuffites with calcareous agglomerates, which are easily weathered and leave various shapes of pits in the longitudinal section of the rocks (at the white arrows). (**c**, **d**) Thin- to middle-bedded tuffites in the middle part of the MHF. (**e**) Longitudinal section of the tuffites, showing small grained bedding in the lower part and horizontal bedding in the upper part.

The middle part of the MHF is a set of highly rhythmic tuffites without carbonate interlayers, locally containing calcareous agglomerates without macrofossils. There are no shelly fossil concentrations in the tuffites. All trilobites are preserved in medium- and thin-bedded silt- to fine-grained tuffites (Figs. 1b–d, 4). The horizontal bedding is developed with no cross-bedding observed. Beds are about 1 cm thick, locally graded, and composed of fine-, silt-, and mud-grained pyroclastic material or ash (Fig. 4e). This evidence suggests that the burial environment experienced had only very weak hydrodynamic energy, and may have formed near or below the storm wave base.

### Co-occurring fossils with *Omegops* sp. A

In the MHF, except for trilobites, other body fossils are very rare. Benthic organisms, including very few brachiopods and tiny crinoid stems, are found sparsely in the medium-grained tuffites of the lower part of the MHF. However, in the middle part of the MHF, where trilobites are more abundant, only isolated trilobites are found and no other benthic taxa are present (Fig. 3a–c). Conversely, some layers of the middle part of the MHF contain small numbers of ammonoids (members of *Gonioclymenia* fauna)^23^, and even co-occurring with the trilobites (Fig. 3d). Radiolarians and sponge spicules can also be observed in rock thin sections.^18^. In addition, some of the layers in the middle part of the MHF contain trace fossils of the blurred *Teichichnus* ichnofabric, which suggests a deeper water environment^24^. However, trace fossils are rare or absent in the beds where trilobites are enriched, reflecting the fact that trilobites are buried in an environment with little bioturbation.

### Comparison with *Omegops* sp. A in the upper part of the LHF

The upper part of the LHF in the same area also contains abundant *Omegops* sp. A in the bioclastic limestones, argillaceous limestones, and shales assemblage (Fig. 1e). The preservation pattern observed in the upper part of the LMF is distinctly different from that of the middle part of the MHF, with the former containing many fossilized carcasses with articulated or enrolled exoskeletons (Fig. 1f) in addition to exuviae in the same beds, and a rough estimate of the ratio of carcasses to exuviae is 1:5 to 1:10; however, this ratio is less than 1:100 in the latter. Further, the fossil assemblages co-occurring with the trilobites in the two members are distinctly different. Other benthic fauna (Fig. 1g), such as brachiopods, corals, bryozoans, and echinoderms^19^, in addition to a large number of carcasses and exuviae of *Omegops* sp. A, are abundant in the upper part of the LHF, but in the middle part of the MHF, these fossils are replaced by ammonites and radiolarians.

## Discussion

Previous work on the spatial distribution of Late Devonian Phacopida trilobites, indicates that *Omegops* favored a relatively shallow-water environment, with a habitat extending from the lower shoreface to upper offshore^25^. This is similar to the depositional environment represented by the upper part of the LHF. Conversely, the middle part of the MHF was deposited in a deeper water, as evidenced by the presence of ammonoids and radiolarians, the lithologic assemblage, and facies markers. The middle part of the MHF likely formed in the lower offshore, near or below the storm wave base, which would have been a deeper environment than the normal habitat of *Omegops*^25^. This observation, combined with the extreme rarity of trilobite carcass fossils (less than 1%) in the middle part of the MHF, suggests that the environment represented by the MHF is likely to be only the molting site of *Omegops* sp. A, rather than their normal habitat. This implies that *Omegops* sp. A may have migrated to a deeper-water environment to molt before returning its shallower habitat, a behavior known as allopatric molting.

Trilobites would have been weak and vulnerable during molting and immediately post molting, when their shells were soft. Therefore, the molting process would have had to occur in a safe environment free from external disturbances, with scarce/no predators or other organisms^1^. Phacopida trilobites were common in the Devonian, but had lost their dominance by the Late Devonian^26^. The presence of a large number of predators (e.g., fish, large arthropods and cephalopods) and competitors in same niche (e.g., brachiopods and corals) undoubtedly exerted strong survival pressure^27^. These pressures would have led to adaptations in behavior, such as an increased tendency to seek shelter, which has been previously observed for some Devonian trilobites^4,28^. Trilobites have also been found to molt in an empty cephalopod shell in the Hongguleleng Formation^5^. *Omegops* sp. A travelling to deeper water to molt may also be shelter-seeking behavior, most directly to avoid predators, such as nautiloids and fish that have been found in same horizon^5,29^.

Trilobites need to molt several times during growth, however, each molt is a fairly brief process. Henningsmoen^1^ speculated that trilobite molting may be completed in only a few minutes based on the molting process of modern decapod crustaceans, and the molting preparation process is only a few hours. The migration distances required for the hypothesis discussed here averages about 40–50 km, with an average slope 0.07°, giving a vertical distance of 50–60 m^30^. It would seem unnecessary for the trilobites to have travelled such long distances to an unfamiliar environment for a molting process requiring only hours. Molting could have been accomplished in a secluded location in their normal habitat, like some modern shrimp and crab species, which seek shelter in plants or rock crevices to molt^1^. Indeed, as mentioned previously, fossil evidence of such molting behavior has been previously described^3–5,31^. There may therefore be other purposes for the allopatric molting behavior that appears to have been preserved by the fossils described in this study.

**Figure 5:**
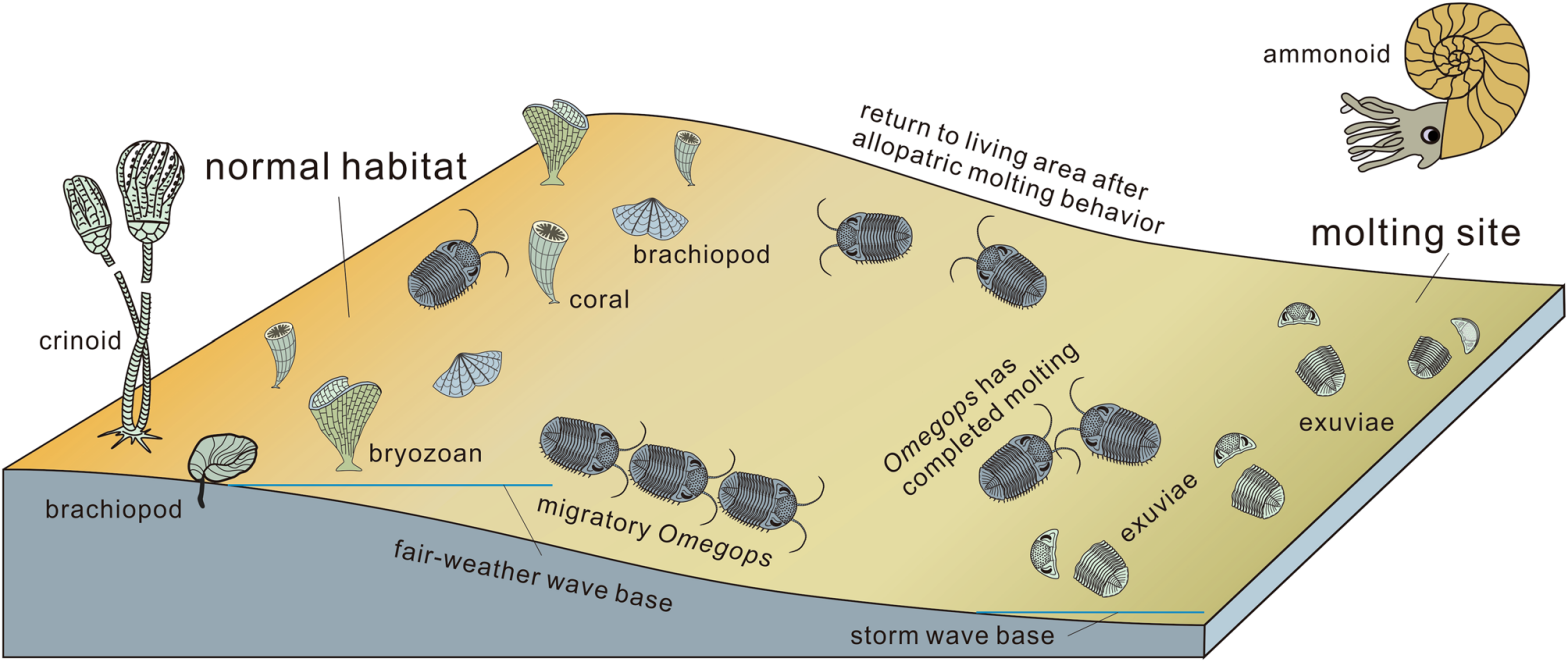
Schematic diagram of the allopatric molting of the Late Devonian trilobite *Omegops* sp. A in western Junggar.

One possibility is that these trilobites in western Junggar needed to adjust their lifestyle during or after molting. Some modern arthropods (e.g., horseshoe crabs, crabs) molt before mating^32^, and some scholars speculated that trilobites may have had similar behavior pattern^33^. If this hypothesis also holds truth for *Omegops* sp. A in western Junggar, it obviously takes longer for them to mate and spawn after molting than for them to molt alone, and the former requires a safer environment, which seems to justify the long journey. Another possibility, as described earlier, is that *Omegops* sp. A molted in deeper-water and then returned to shallower habitat (Fig. 5), it is also a kind of migration behavior in addition to reflecting the allopatric molting. Besides trilobites, a large number of brachiopods, echinoderms, corals and bryozoans were found in the Late Devonian Famennian strata in the study area, their abundance and diversity are much higher than that of trilobites^19,34,35^. The presence of these species inevitably leads to a reduction in the living space of trilobites. As a movable benthic organism, trilobites may have been trying to find a new or temporary living space by migrating to deeper water for molting. Compared with shallow-water, deeper water is differently affected by environmental impacts, and shallow-water organisms have been disproportionately affected during some extinction events^36^. Deeper water habitats could even have provided a temporary refuge for some organisms^37^. There are also cases of species migrating from shallow to deep water during the geological period^38^. The migration of the Late Devonian *Omegops* sp. A in western Junggar to deeper water for molting may represent an attempt by these trilobites to expand their ecological domain into deeper water, which suggests that migration to deeper water may have been a survival strategy for trilobites coping with an crowded environment, or an environment with multiple predators.

## Conclusions

The *Omegops* sp. A found in the middle part of the MHF (Upper Devonian) in western Junggar are almost exclusively in situ exuviae, while carcass fossils are extremely rare. Based on this preservation pattern, the burial environment, and the set of organisms co-occurring with these trilobites, we suggest that the environment represented by the middle part of the MHF was the molting site for *Omegops* sp. A, rather than their normal habitat. This interpretation is supported by previous work suggesting that *Omegops* primarily lived in a lower shoreface to upper offshore environment^25^. We suggest that this species of trilobite had the behavior of allopatric molting, in which individuals travelled to deeper water to molt. The main reason for the appearance of this behavior may have been to avoid adverse ecological factors such as predators and competitive organisms. This implies the existence of migratory behavior among Devonian trilobites and indicates that Devonian trilobites actively expanded to deeper water or find new ecological domains when faced with unfavorable ecological environment.

## Data Availability

All data generated or analyzed during this study are included in this published article.
